# Electroencephalography-Based Neuroemotional Responses in Cognitively Normal and Cognitively Impaired Elderly by Watching the *Ardisia mamillata Hance* with Fruits and without Fruits

**DOI:** 10.3390/ijerph191610020

**Published:** 2022-08-14

**Authors:** Juan Du, Xiaomei Chen, Li Xi, Beibei Jiang, Jun Ma, Guangsheng Yuan, Ahmad Hassan, Erkang Fu, Yumei Huang

**Affiliations:** 1 College of Landscape Architecture, Sichuan Agricultural University, Chengdu 611130, China; 2 Maize Research Institute, Sichuan Agricultural University, Chengdu 611130, China

**Keywords:** EEG, *Ardisia mamillata Hance*, elderly, fruit plants, neural emotion

## Abstract

Background: The purpose of this study is to explore the difference between the indexes of neuro-emotion between the cognitively normal elderly (CNE) and cognitively impaired elderly (CIE) while viewing the *Ardisia mamillata Hance* with red fruit (F) and without red fruit (NF) to determine which kind of the *Ardisia mamillata Hance* would be more beneficial to the participants’ neuro-emotions. Methods: Nine CNE individuals and nine CIE individuals, ranging in age from 80–90 years old, participated in this study and signed the informed consent form before beginning the experiment. Six mood indicators (engagement, excitement, focus, interest, relaxation, and stress) were measured by an EEG headset during the participants’ viewing of the NF, F, and NF + F. Results: For the CNE group, their engagement, excitement, and focus values were the lowest, while their interest value was the highest when they view the NF + F; therefore, we obtain the results that the combination of NF + F was the most beneficial to their EEG emotions. For the CIE group, the combination of NF + F increased their interest score, but decreased their focus score, which indicated that the NF + F was the most beneficial to their neuro-emotions. Conclusions: This study concluded that the combination of plants with and without fruits was most beneficial to the neural emotions of both groups of elderly people. Especially for the CIE, plants with larger and warmer colors, such as yellow, red, and orange fruits, should be considered for installation indoors or outdoors, as this would be better for their emotional well-being.

## 1. Introduction

### 1.1. Current Situation of Population Aging

At present, the perils of aging in China are severe, and the aging process is accelerating [1]. By 2050, China’s elderly population over 65 years of age is expected to approach one-third of the total population [2]. This has led to a significant increase in the prevalence of physical and mental diseases associated with advanced age [3]. The main diseases suffered by the elderly in China include: physical health disorders caused by unhealthy lifestyles, such as cancer, cardiovascular diseases, stroke, and obesity; as well as mental health disorders, such as dementia and depression [3,4]. According to current evidence, the rate of cognitive impairment among the elderly in China is 22.0% [5], and the rate of dementia in the elderly aged 60 and above is 18.7% [6]; it is predicted that there will be a continuous upward trend in the next 30 years [7]. In addition to physical and psychological stressors, the elderly population also faces economic stressors as they age [8].Several social surveys show that in addition to material assistance from society, the elderly also need more psychological and spiritual attention [9]. In order to promote healthy aging of the population, it is necessary to increase the publicity regarding popular science of mental health among the entire population, focusing on the psychological problems of key groups such as the elderly, specifically to bolster the prevention of Alzheimer’s disease [10].

### 1.2. Effects of Plants on Physical and Mental Health of the Elderly

It has long been proved that there is a close connection between health and the natural environment [11]. According to Wilson’s Biophilic Theory [12], a good relationship between man and nature is the foundation of physical and mental health. Ulrich believes that natural elements can influence people’s perception, emotion, and even cognition through their unconscious or conscious mind, and finally, promote stress relief [13,14]. Plants are an important part of nature, and all forms of life, including humans, are intrinsically related to plants [15]. Plants can not only release a large number of negative oxygen ions, resulting in beneficial effects such as the purification of the air, air temperature regulation, and dust and noise reduction, but also to improve and promote the physical and mental health of the elderly [16,17]. Sensory function is a physiological skill for humans to perceive stimuli in the surrounding environment, and it is a bridge between human subjective feelings and the external objective environment [18].The sensory functions of the elderly usually decline in the order of sight, hearing, smell, taste, and touch as they age, so multisensory environmental interventions have become one of the non-pharmacological treatment options for various diseases in the elderly [19]. The health effects of plants on the elderly are usually achieved with sensory stimulation.

The health effects of plants on the elderly are usually achieved through sensory stimulation, and current research mainly focuses on the sensory stimulation of vision, smell, and touch of the elderly. In terms of visual sensory stimulation, Hassan et al. [20]. found that older adults had lower systolic blood pressure and lower anxiety scores after viewing the money tree plant for 5 min compared to a control group without plants, indicating effective mental and physical relaxation. Chia-Pin Yu et al. [21] reported that after viewing plants in a forest bath, 128 middle-aged and older adults had significantly lower pulse, diastolic, and systolic blood pressure, as well as significantly lower anxiety levels. Xiu Meiling [22] found that when looking at green, red, and purple morning glory, the elderly male individuals had lower blood pressure and arterial pressure when viewing the green morning glory, while the elderly women had no obvious physiological changes in response to the three colors of morning glory. In terms of olfactory sensory stimulation research, Khondzadeh et al. [23] reported through a controlled experiment that the cognitive function of Alzheimer’s patients was improved after 4 months of smelling lemon balm, and it also had a positive effect on agitation. Scholars such as Heng L [24] and Xiaos L [25] have demonstrated through experiments that plant essence helps improve hypertension, asthma, and poor sleep in the elderly. Research on tactile sensory stimulation is often carried out in conjunction with hands-on horticultural therapy (HT) activities, and both Andrea’s and Wei’s studies have shown that the practice of plant cultivation can improve stress levels and restore physical function in affected elderly people [26,27]. Thaneshwari et al. [28] recommend that therapeutic gardens be designed specifically for the care of certain types of patient, such as Alzheimer’s gardens. Therapeutic gardens should be used to improve the health and wellbeing of dementia patients [29]. A quantitative review by Zhao et al. [30] on the benefits of gardening for people with dementia reported improvements in cognitive function, agitation, emotional state, and engagement. The 24-session gardening program significantly improved cognitive function and brain-derived neurotrophic factor levels related to cognitive health factors of the participating elderly [31]. One course of gardening (four weeks, once a week) can effectively improve the mental and physical health of the elderly with dementia [32]; HT can improve depression and agitation in dementia patients [33,34,35,36].

### 1.3. Electroencephalography (EEG)-Based Neural Emotion

Emotion is a reflection of human perception and awareness of various things, and it is closely related to people’s physical, mental, and spiritual health [37]. There are more and more scholars studying the relationship between emotions and health [38,39,40]. Emotions are closely related to the cerebral cortex and the limbic system, and their physiological basis is the central nervous system; when people are in different emotional states, the state and activities of the brain are different [41,42]. In the human brain, tens of billions of neurons are active [43]. When neurons transmit bioelectrical signals, they produce waves called brain waves, or EEG signals. EEG is a method of collecting data from brain waves through sensors [44]. Among the many methods to study brain activity, EEG is increasingly used by scholars to study brain activity and identify emotions due to its low cost, simplicity, and non-invasiveness [45,46,47].

The frequency of the EEG signal is generally between 0–50 Hz, and the EEG signal can be divided into five bands according to frequency: δ band (0.5–4 Hz), θ band (4–8 Hz), α band (8–14 Hz), β band (15–30 Hz), and Y band (30–50 Hz) [48]. Xing M et al. [49] investigated the cognitive load during emotion regulation using EEG data and found that the θ band feature path length values increased during emotion regulation, and thus the theta band was considered to be particularly important for emotion recognition. Kim et al. [50] used EEG to compare the effects of gardening and non-gardening activities on cognitive function in the elderly, and the results reported an increase in prefrontal beta and gamma indices when the elderly performed gardening activities, indicating improved cognitive function in the elderly. Tao J et al. [51] used EEG to obtain brainwave data to explore the effects of flower basket making activities on human physical and mental health, and their results showed that α brainwave values increased during gardening operations, which could be used to describe the participants’ relaxation state. In addition, the detection of emotions by EEG can be analyzed by using six emotional indicators (stress, engagement, interest, excitement, concentration, and relaxation). Chengc Z et al. [52] used the six neuroemotional indicators of EEG to study the effects of visual density and integrated acoustic environment on humans in green space. Juan D et al. [53] also used six emotional indicators of EEG to study the neuroemotional responses of normal elderly and cognitively impaired elderly to flower arranging, and found that both groups achieved relaxation, especially the cognitively impaired elderly, who showed an interest in flower arranging.

**Figure 1:**
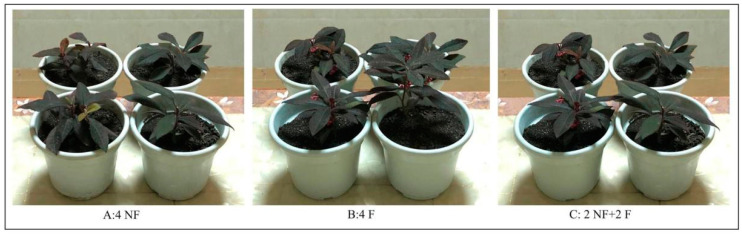
*Ardisia mamillata Hance* (**A**): 4 NF, (**B**): 4 F, (**C**): 2 NF + 2 F.

Although there are numerous studies demonstrating the health effects of plants on the elderly, no studies have been reported on the emotional effects of fruit plants on the cognitively normal elderly (CNE) and cognitively impaired elderly (CIE). In this study, we selected the *Ardisia mamillata Hance* as the plant material. Its leaves remain green during all seasons, and the red fruits can also last for eight months, so it is an excellent leaf- and fruit-watching plant (Figure 1). The questions to be answered by this study were: (1) What are the differences in the indexes of neuro-emotion between the CNE and CIE in viewing the *Ardisia mamillata Hance* with and without fruits? (2) Which kind of the *Ardisia mamillata Hance* would be more beneficial to subjects’ EEG-based neuro-emotion? The results of this study can provide a reference for the design of indoor and outdoor plant landscapes for the elderly.

## 2. Materials and Methods

### 2.1. Participants

Volunteer participants came from the senior housing in Wenjiang County, Chengdu City, China. More than 40 elderly people live in this housing facility, which has more than 60 rooms, with each elder having one room in which to live. We test the cognitive ability of participants with the mini-mental state examination (MMSE). According to the Chinese version of the MMSE [54] and considering the educational background of the participants, the definition of cognitive impairment was: no education, ≤17 points, primary school education, ≤20 points, and secondary education or higher education, ≤24 points. A total of 9 CNE and 9 CIE, ranging in age from 80–90 years old, were selected by the MMSE (mini-mental state examination) as participants, and they signed the informed consent form before beginning the experiment. This study was conducted with the approval of the local ethics committee of the College of Sichuan Agricultural University, China (AN20210160). This experiment was carried out on 10–14 April 2021, 9:00–11:30 a.m. and 14:00–17:30 p.m., in a quiet 3.6 m wide and 6 m long indoor room in senior housing; the illumination, humidity, and temperature of room were maintained at 500 lux, 50–55%, and 22–25 °C, respectively.

### 2.2. Materials and Equipment

#### 2.2.1. Plant Materials

*Ardisia mamillata Hance* has dark green leaves, red berries, and unscented fruit that is not edible. It is a shade tolerant plant that can be kept indoors for viewing. In this experiment, the *Ardisia mamillata Hance* with the fruits (F) and without fruits (NF), as well as the combination of NF + F were used as the observation materials, as shown in Figure 1: 4 NF, 4 F, and 2 NF + 2 F (2 NF were from 4 NF; 2 F were from 4 F).

#### 2.2.2. Equipment

**Figure 2:**
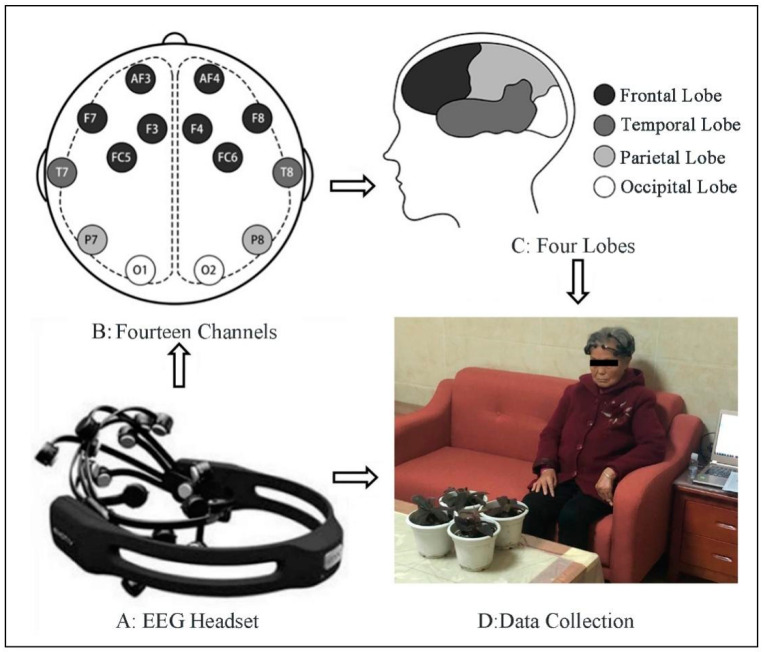
EEG Data Collection.

We used the EPOC + EEG headphones to test the particiipants’ indexes of neuro-emotion (Figure 2A). EEG neuroemotion in this study refers to the six emotional indicators from the brain identified by EEG: engagement, excitement, focus, interest, relaxation, and stress. This research refers to the previous study [55] using the EEG headset, particularly Emotiv’s explanation, to present the working definitions of each emotional parameter: “engagement” reflects the degree of immersion, investment or attraction, “excitement” represents the level of arousal of the individual and is associated with a high degree of arousal. “Focus” is a high state of arousal, reflecting high attention. “Interest” indicates the degree of liking or disliking the current environment, which represents the attractiveness of the environment. “Relaxation” is associated with low levels of arousal and relaxation; when people are in a relaxed state, their heart rate slows, their blood pressure drops, and their level of arousal decreases. “Stress” refers to the psychological tension reaction or state formed under the action of individual anxiety or fear, which represents the negative valence dimension of people’s emotions, such as frustration and disappointment.

Four cerebral lobes (frontal occipital lobe, temporal lobe, parietal lobe, occipital lobe) of the participants were tested using the 14 electrodes (O1, O2, AF3, F3, AF4, F4, FC5, FC6, F7, P7, T7, F8, T8, P8) of the headphones (Figure 2B,C). The frontal lobe (F3, F4, AF3, AF4) mainly reflects the emotion, language, cognition, and behavior of the brain. The temporal lobe (T7, T8) mainly controls language processing and audio-visual memory, the parietal lobe (P7, P8) mainly controls reception and sensory connection, and the occipital lobe (O1, O2) mainly reflects all visual information of the brain. Six emotional indexes to reflecting emotional change from the brain activities were recorded, one time each minute, by EEG, and these were transmitted to a computer via a Bluetooth signal (Figure 2D).

### 2.3. Procedure

**Figure 3:**
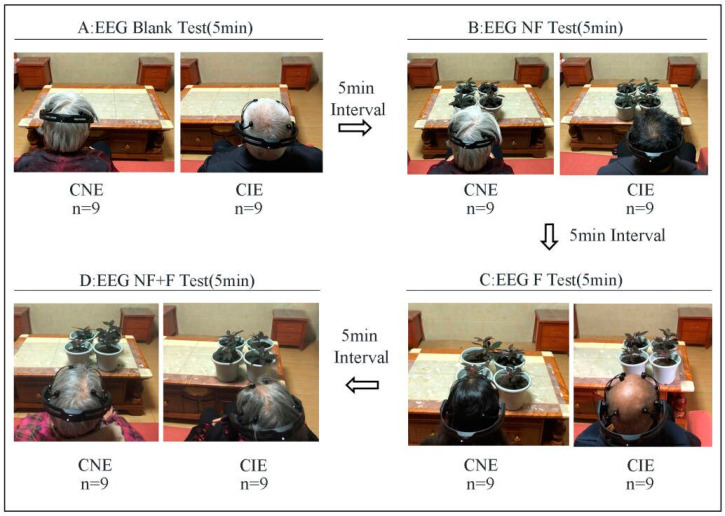
Study procedure (**A**): EEG Blank Test. (**B**): EEG NF Test. (**C**): EEG F Test. (**D**): EEG NF + F Test.

All participants took part in the experiment in sequence, entering the room one by one. Before the test, all the participants learned the experimental process and were told calm their brains with 5 min of sitting quietly. In the pres-test, the blank test was carried out firstly facing a table without any plant (Figure 3A). After that, the plants (4 NF, 4 F, 2 NF + 2 F) were placed on the table in front of the subjects in turn, and six emotional indexes were output by the EEG headset once a minute; each test took 5 min. The interval time of each test was 5 min for the participants (Figure 3B–D). In each test, each emotion indicator was collected five times, and the averaged value was calculated as the final test value.

### 2.4. Statistical Analysis

The data was analyzed using SPSS 24.0 (SPSS, Armonk, NY, USA) software. For the data that met the normal distribution, we used a paired *t*-test, while for the data that did not meet the normal distribution, we used the Wilcoxon signed-rank test.

## 3. Results

**Figure 4:**
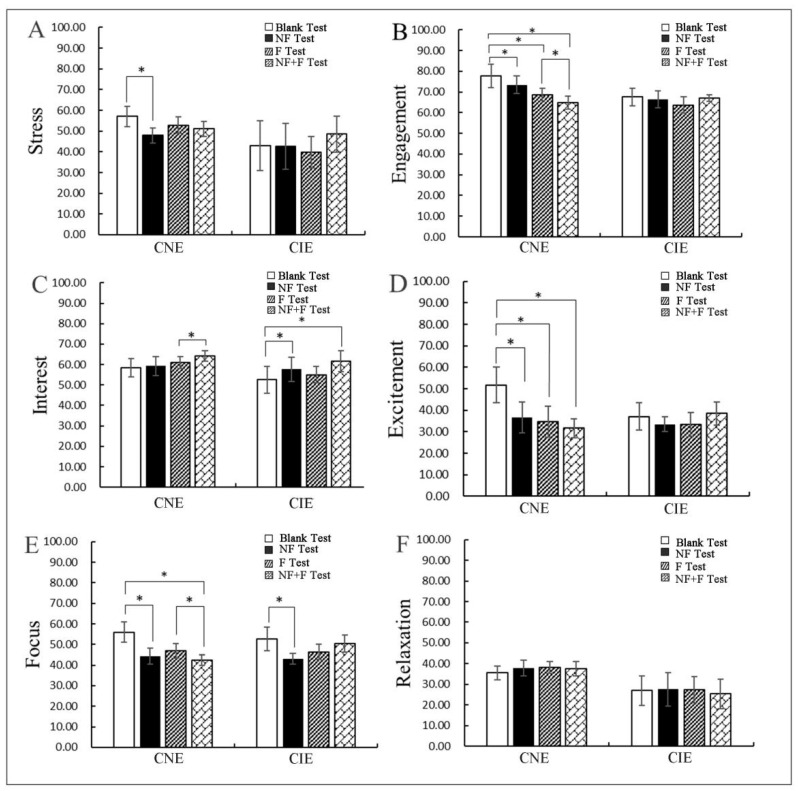
The analysis of the difference in significance between the tests (* sig. < 0.05); (**A**) stress, (**B**) engagement, (**C**) interest, (**D**) excitement, (**E**) focus, (**F**) relaxation.

In the stress test (Figure 4A), the results showed a significant difference (*t* = 2.409, sig. < 0.05) between the blank test and NF test in the CNE group. The stress value of the CNE significantly decreased in the NF test comparing with the blank test. The reduced stress in the CNE indicated that the participants were at a relaxed stage while viewing the plants. For the CIE, stress levels did not change significantly between each test. Therefore, the CNE group had less stress with NF. 

In the engagement test (Figure 4B), significant differences existed in these pairwise tests, including blank test and NF test (z = −1.988 ^b^, sig. < 0.05) (Wilcoxon signed-rank test), blank test and F test (*t* = 2.256, sig. < 0.05), blank test and NF + F test (*t* = 2.73, sig. < 0.05), F and NF + F test (*t* = 2.609, sig. < 0.05) in the CNE group. The engagement value of the CNE continued to decline when viewing different plants, and the lowest engagement value existed in NF + F test. However, no significant difference existed between the each test in the CIE group. Therefore, the CNE group had lowest engagement with NF + F.

In the interest test (Figure 4C), there was a significant difference between the NF test and NF + F test in the CNE group (z = −2.193 ^c^, sig. < 0.05) (Wilcoxon signed-rank test), while the significant differences shown in these tests were for the blank test and NF test (*t* = −2.911, sig. < 0.05), and the blank test and the NF + F test in the CIE group (*t* = −2.989, sig. < 0.05). These results showed that the interest value of the two groups increased in the NF + F test. This means that both groups showed the highest interest value in the NF + F test. Therefore, both groups showed the highest interest with NF + F.

The results of the excitement test (Figure 4D) showed that there were significant differences between the blank test and other tests, including the blank test and NF test (z = −2.090 ^b^, sig. < 0.05) (Wilcoxon signed-rank test), the blank test and the F test (*t* = −2.809, sig. < 0.05), and the blank test and the NF + F test (*t* = 2.877, sig. < 0.05) in the CIE group. The excitement value of the CNE continued to decline in each test, and the lowest value was in the NF + F test, but the excitement value of CIE showed little change in each test. Therefore, the CNE group showed the lowest excitement value with NF + F.

The results of the focus test (Figure 4E) showed that the significant differences were observed between the blank test and other tests, including the blank test and NF test (z = −2.090 ^b^, sig. < 0.05) (Wilcoxon signed-rank test), and the blank test and NF + F test (*t* = 2.504, sig. < 0.05) in the CNE group. The focus value of the CNE continued to decline in the post-test, and the lowest focus value was in the NF + F test. While the lowest focus value of CIE was in the NF test, when compared with the blank test, there was a significant difference (*t* = 2.559, sig. < 0.05). Therefore, the CNE group had lowest focus value with NF + F, while the CIE group had the lowest focus value with NF. 

The results of the relaxation test (Figure 4F) showed that no significant difference existed between each test in both groups.

## 4. Discussion

The differences regarding the indexes of neuro-emotion between the CNE and CIE in viewing the *Ardisia mamillata Hance* with fruits (NF) and without fruits (F) were presented in this study.

Firstly, there were significant differences in stress (Figure 4A), engagement (Figure 4B), excitement (Figure 4D), and focus (Figure 4E) for the CNE group between the blank test and NF test; their stress, engagement, excitement, and focus values in the NF test decreased significantly compared with blank test. “Stress” refers to the psychological tension reaction or state formed under the action of individual anxiety or fear, which represents the negative valence dimension of people’s emotions, such as frustration and disappointment. “Engagement” can reflect the degree of immersion, investment, or attraction, “excitement” represents the level of arousal of the individual and is associated with a high degree of arousal, “focus” is a high state of arousal, reflecting high attention [55]. Therefore, the decrease in stress indicated that the CNE had less stress, the decrease in engagement indicated that the CNE were in state of low participation and low immersion (which was a state of relaxation), the decrease in excitement indicated that the CNE tended to be calm, decreased focus reflects the appearance of low attention, indicating that the CNE were in a relaxed state. The results show that the CNE were less stressed and in a more relaxed and calm state with plants than without the plants.

While significant differences in interest and focus were observed for the CIE group between the blank test and NF test, their interest value increased and their focus value decreased in the post-test. “Interest” indicates the degree of liking or disliking the current environment, which represents the attractiveness of the environment [55]. The increased interest suggested that the CIE prefer environments with plants, and the decreased focus reflects the appearance of low attention, indicating that the CIE were also in a relaxed state. Therefore, we see that the CIE were more relaxed with plants than without plants. Previous studies [20,21,22] had confirmed that the plant-based environment lowered the subjects’ blood pressure, reduced their anxiety, and promoted their emotional relaxation. Actual plants make people feel more relaxed and emotionally stable compared to the absence of plants [32,56,57,58]. This was consistent with the results of this study.

Secondly, significant differences were observed for engagement (Figure 4B) and excitement (Figure 4D) in the CNE group between the F test and blank test. Their engagement and excitement values continued to decrease; this showed that they were less engaged and less excited, while watching the F calmed them more when compared with the blank test. However, there were no significant differences in neuro-emotional values in the CIE group between the F test and blank test, or between the F test and NF test. Elderly people’s vision, hearing, smell, taste, touch, and so on are gradually declining [19]. In this experiment, the fruit of *Ardisia mamillata Hance* was relatively small, about 1 cm in diameter, and hidden in the leaves, which made it difficult for the CIE to watch, and as we know, especially for the CIE, cognitive impairment caused by neuropathy in the brain can lead to deterioration in language, visuospatial ability, and attention [59,60,61]. Their impaired vision and cognitive impairment would have made this index even more difficult, so there was no significant change in their emotions when watching these two groups of plants. Therefore, for the CIE, in order for them to more easily observe the fruit, it would be necessary to use a plant, either indoors or outdoors, with a larger fruit size. 

Thirdly, for the CNE, their engagement, excitement, and focus values were the lowest during viewing the NF + F; referring to the definitions of these three indicators [55], we believe that they were most relaxed with the NF + F. However, the interest value was the highest for the CIE with the NF + F, which indicated that this combination of plants engendered the greatest interest, so the combination of the NF + F was more beneficial to their neuro-emotion not only for the CNE, but also for the CIE. When designing plant landscaping for the elderly, we should consider combining fruit plants and leaf plants. Furthermore, Wang et al. [62] reported that chromatic and achromatic colors led to different relaxation responses. Dong X et al. [63] found that colorful plants can stimulate vison and improve sleep quality in CIE people, as well as reduce restless behavior. Choi Y et al. [64] suggested that the elderly prefer warm colors such red, yellow, and so on, so we suggest that these fruit plants with bright warm colors such as yellow, red, and orange fruits should be the focus when choosing indoor or outdoor fruits plant for the elderly; this is more conducive to promoting the healthy mood of the elderly. 

Finally, some limitations of this study exist. The sample size was too small; therefore, in future research, we hope to expand the number of subjects to collect more data. The fruit of *Ardisia mamillata Hance* was too small; in future experiments, it is suggested to choose fruit plants with larger fruits, so as to facilitate observation by the elderly. In addition, different colors and fragrances of fruit plants, as well as different gender, can be considered for future research.

## 5. Conclusions

This study chose to explore the differences regarding the indexes of neuro-emotions between the CNE and CIE in viewing the *Ardisia mamillata Hance* with fruits and without fruits and which kind of the plants would be more beneficial to their neuro-emotions. For the CNE, their engagement, excitement, and focus values were the lowest while their interest value was the highest when viewing the NF + F; therefore, we found that the combination of NF + F was most beneficial to their EEG emotions. For the CIE, the combination of NF + F increased their interest score, but decreased their focus score, indicating that the NF + F was the most beneficial to their neuro-emotions. Finally, this study concluded that the combination of plants with and without fruits was the most beneficial to the neural emotions of both groups of elderly people. Especially for the CIE, plants with larger and warmer colors, such as yellow, red, and orange fruits, should be considered to install indoors or outdoors, as this would be better for the emotional well-being of the elderly.

## Data Availability

Not applicable.
